# CD34‐Positive Acral Chondromyxoid Mesenchymal Neoplasm Harboring a Novel *TCF4::ERG* Fusion

**DOI:** 10.1002/gcc.70073

**Published:** 2025-08-12

**Authors:** Eric C. Honaker, Laura M. Warmke, Ameline Baptiste, Daniel Baumhoer, Esther Baranov, Eitan Halper‐Stromberg, Carina A. Dehner

**Affiliations:** ^1^ Department of Pathology and Laboratory Medicine Indiana University School of Medicine Indianapolis Indiana USA; ^2^ Bone Tumor Reference Center at the Institute of Medical Genetics and Pathology University Hospital and University of Basel Basel Switzerland; ^3^ Basel Research Centre for Child Health Basel Switzerland; ^4^ Department of Pathology and Laboratory Medicine University of Pennsylvania Philadelphia Pennsylvania USA

**Keywords:** acral chondromyxoid tumor, CD34‐positive, ERG, mesenchymal tumor, TCF4

## Abstract

Molecular testing has significantly transformed the field of anatomic pathology over the past several decades. Despite these advances, acral mesenchymal neoplasms remain diagnostically challenging, requiring careful integration of clinical presentation, histologic features, and molecular findings for accurate classification. Herein, we present a case of an acral chondromyxoid mesenchymal neoplasm harboring a novel in‐frame *TCF4::ERG* fusion involving the right index finger of a 26‐year‐old female. Morphologically, this tumor consisted of nests and sheets of monotonous small round‐to‐ovoid cells embedded in a background of chondromyxoid stroma and hyalinized collagen. The tumor cells were diffusely CD34, ERG, and focally p63 reactive, while S100 protein, cytokeratin AE1/AE3, Pan‐TRK, ALK, smooth muscle actin, and desmin were negative. Albeit short follow‐up (3 months), the patient continues to do well without evidence of metastasis or local recurrence.

## 1 Introduction

Acral mesenchymal neoplasms represent a heterogeneous group of tumors arising from soft tissues in distal anatomical sites, such as the hands and feet. These neoplasms encompass a broad diagnostic spectrum ranging from benign to malignant entities, and they often pose diagnostic challenges due to overlapping histologic features and unique site‐specific considerations. We recently encountered an unusual acral chondromyxoid mesenchymal neoplasm with a novel *TCF4::ERG* fusion during routine sign‐out. We herein report our experience with this distinctive neoplasm to raise awareness and illustrate the key morphologic differential diagnoses.

## 2 Materials and Methods

### 2.1 Case Identification

The case was seen during routine sign‐out on the institutional Bone and Soft Tissue Pathology service. Information regarding clinical presentation was obtained by the primary pathologist from the requisition and the electronic medical record system.

### 2.2 Immunohistochemistry

Immunohistochemistry was performed on formalin‐fixed paraffin‐embedded tissue using the Dako Omnis instrument by Agilent Technologies Inc. for detection of the following antigens: ERG (Dako, clone EP111, RTU), CD34 (Dako, clone QBEnd10, RTU), p63 (Dako, clone DAK‐P63, RTU), Pan‐TRK (Abcam, clone EPR17341, dilution 1:500), desmin (Nordic Biosite, clone BS21, dilution 1:800), pankeratins (AE1/AE3 cocktail, Dako, RTU), ALK (Cell Signaling, clone D5F3, dilution 1:50), smooth muscle actin (Dako, clone 1A4, RTU), and S100 (Dako, clone Poly, RTU). All control slides stained appropriately.

### 2.3 RNA Sequencing

The specimen was sent to Tempus AI Inc. (Chicago, IL) for RNA sequencing. Per the report assay description, chromosomal rearrangements and altered splicing events were detected by hybrid capture next generation sequencing which utilized an IDT xGen Exome Research Panel v2 probe set that consists of more than 415 000 probes and spans 34 Mb target regions within the human genome.

## 3 Results

### 3.1 Case Report

A 26‐year‐old female with a family history of tenosynovial giant cell tumor presented with a palpable mass near the pulp volar of her right index finger. She first noticed the mass 3–5 years prior; however, it became increasingly bothersome, and she desired removal. The mass was excised, and post‐operative computed tomography (CT) imaging of the chest, abdomen, and pelvis, as well as whole body Positron Emission Tomography (PET), demonstrated no evidence of metastatic disease. Three months after excision, the patient is doing well and undergoing active surveillance, with plans for possible re‐excision for definitive management.

### 3.2 Microscopic Findings

**FIGURE 1:**
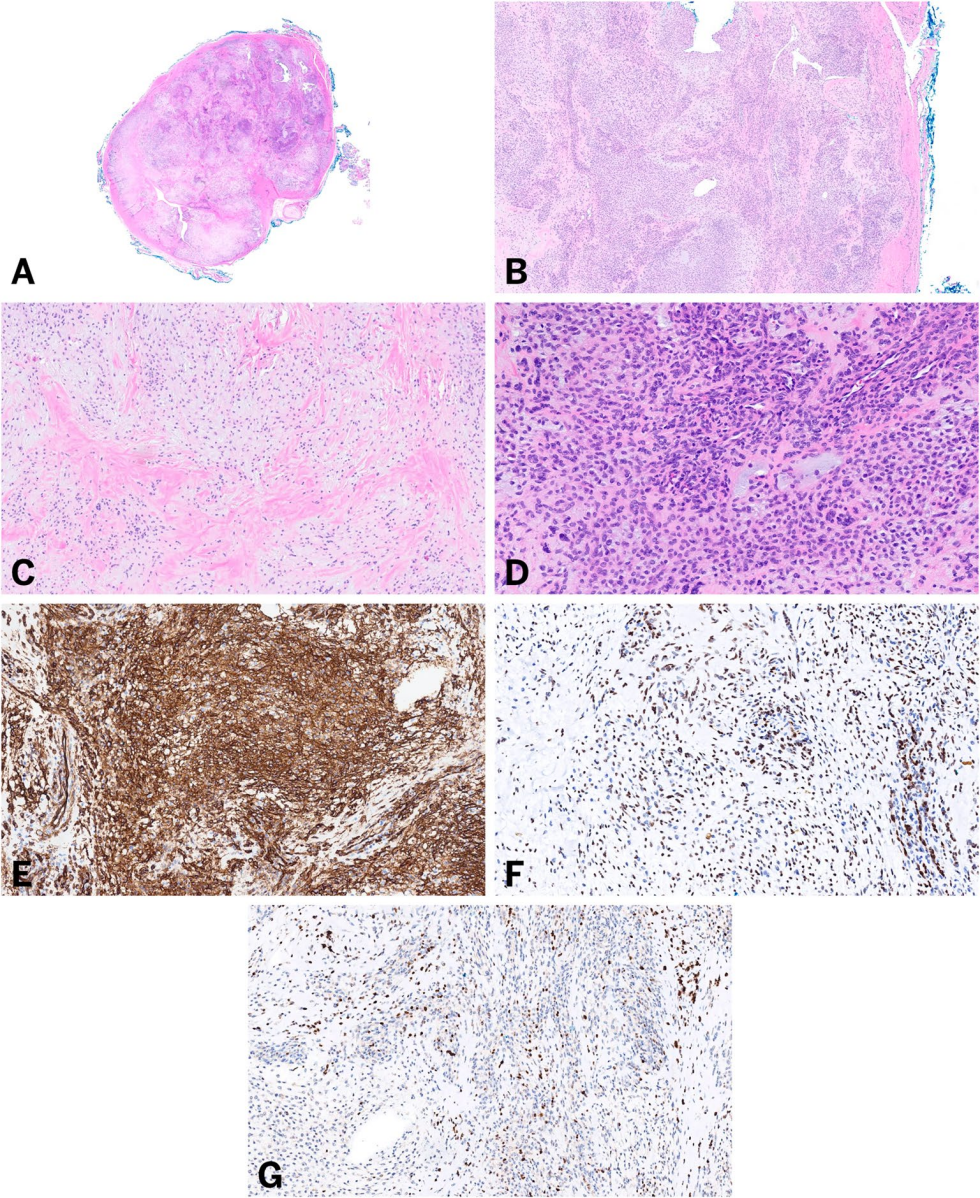
Morphologic features of an acral chondromyxoid mesenchymal neoplasm harboring a *TCF4::ERG* fusion. Low magnification (A, Hematoxylin and Eosin, 1×) and intermediate magnification (B, Hematoxylin and Eosin, 4×) show sheets and nests of small round‐to‐ovoid cells with surrounding chondromyxoid stroma and cystic change. High power examination (C, Hematoxylin and Eosin, 10× and D, Hematoxylin and Eosin, 20×) demonstrates the monotonous morphology of the tumor cells with hyalinized collagen. Strong CD34 (E, 20×) and ERG (F, 20×) immunohistochemical reactivity within the lesional cells. (G, 20×) Focal p63 reactivity.

Grossly, the specimen was described as a single well‐defined nodule. Histologic sections showed a mesenchymal neoplasm, predominantly composed of monotonous small round‐to‐ovoid cells in a distinctive nested and sheet‐like growth pattern with surrounding chondromyxoid stroma (Figure 1A–D). Additionally, hyalinized collagen and abundant cystic change were noted. The lesional cells demonstrated strong and diffuse immunohistochemical reactivity with both CD34 and ERG, and focal reactivity with p63 (Figure 1E–G). Immunohistochemistry for Pan‐TRK, desmin, cytokeratin AE1/AE3, ALK, smooth muscle actin, and S100 protein was negative. The lesion involved the excision margins.

### 3.3 Molecular Findings

**FIGURE 2:**
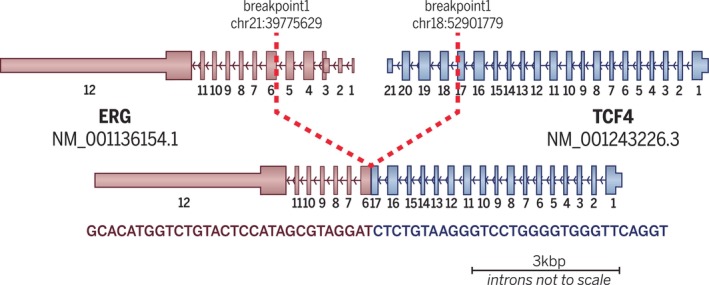
Diagrammatic representation of the in‐frame *TCF4::ERG* gene fusion detected by RNA sequencing, including a demonstration of the location of each gene on their respective chromosomes with exonal breakpoints.

Given the unusual morphology and the uniform appearance of the tumor cell population, RNA‐based molecular testing was performed to investigate the presence of a gene fusion. Analysis by Tempus AI Inc. identified a recurrent in‐frame *TCF4* exon 17:*ERG* exon 6 fusion (Figure 2).

## 4 Discussion

*ERG* (ETS‐related gene) is localized to chromosome 21q22 and is a member of the erythroblast transformation‐specific (ETS) family [1]. *ERG* encodes a transcription factor which participates in many physiologic functions including angiogenesis, hematopoiesis, neuronal development, embryonic formation of the urogenital and vascular systems, and cellular proliferation, migration, permeability, and apoptosis [2]. *ERG*‐related fusions have been identified in a variety of tumors, such as Ewing sarcoma (i.e., *FUS::ERG* and *EWSR1::ERG*) [3], prostatic adenocarcinoma (*TMPRSS2::ERG*) [2], dermatofibrosarcoma protuberans (*MAP3K7CL::ERG*) [4], and a variety of acute leukemias [5].

In contrast, the fusion partner *TCF4* gene is located on chromosome 18q21.2 and encodes up to 18 unique proteins, including transcription factor 4, which is a class I protein within the basic helix–loop–helix (bHLH) transcription family [6, 7]. While fusions involving *TCF4* have been reported in several cases of acute lymphoblastic leukemia/lymphoma [8] and brain tumors [9], its relevance in cutaneous and deep mesenchymal tumors remains unclear.

To the best of our knowledge, this is the first case of an acral chondromyxoid mesenchymal neoplasm with a *TCF4::ERG* fusion. While MacKeracher et al. briefly mentioned a “mesenchymal neoplasm NOS” on the fifth finger in their supplemental material, no histology was provided to compare to the current case [10]. It is certainly worth speculating that the acral location in conjunction with the identical fusion would point toward a possible shared histogenesis (and morphology). However, until additional data on such tumors becomes available, the natural history of this neoplasm remains unclear.

The list of acral mesenchymal neoplasms to consider is long; the histologic and immunohistochemical similarities between hyalinizing and chondromyxoid tumors on acral sites can be diagnostically challenging.

For instance, the recently described *EWSR1::SMAD3* rearranged fibroblastic tumor shares some morphologic features with the present case as it is characterized by hypocellular hyalinized areas coalescing with hypercellular areas of spindled fibroblasts and is also known to demonstrate strong diffuse nuclear expression of ERG by immunohistochemistry [11, 12]. However, this tumor is typically CD34 negative and defined by its unique fusion [11, 12].

Another uncommon acral tumor is the *OGT*‐rearranged acral mesenchymal neoplasm [13, 14]. This exceedingly rare tumor is characterized by hypocellular regions consisting of hyalinized or myxoid stroma and areas of hypercellular epithelioid cell proliferation [13, 14]. Reports of this tumor show focal CD34 and epithelial membrane antigen (EMA) reactivity [13].

Another chondromyxoid tumor with an acral predilection, which most commonly arises in the subungual or periungual regions, is superficial acral fibromyxoma (SAF) [15, 16, 17]. SAFs are composed of a poorly marginated population of stellate‐to‐spindled cells arranged in a loosely fascicular pattern embedded within alternating dense hyaline cartilage and myxoid stroma [16, 17]. While not required for the diagnosis of this tumor, loss of *RB1* is supportive [15]. SAFs demonstrate CD34 reactivity (approximately 69%–90% of reported cases) and are typically pankeratin negative [16, 17]. However, SAFs also show variable and usually focal reactivity for S100 protein, EMA, and smooth muscle actin [15, 16, 17].

Given the focal p63 expression, cutaneous myoepithelioma was briefly considered. Round‐to‐ovoid cell morphology in cutaneous myoepitheliomas can occasionally be seen, but more commonly, these demonstrate a lobular architecture with nests of plasmacytoid epithelioid cells with surrounding myxoid stroma [18, 19]. The co‐expression of a neural and an epithelial marker, such as S100 protein and cytokeratin AE1/AE3, respectively, is usually required for this diagnosis [18, 19]. Further, about 50% of cases harbor recurrent fusions involving the *EWSR1* gene [20, 21].

Acral fibrochondromyxoid tumor, another emerging entity, which harbors a characteristic *THBS1::ADGRF5* fusion, is also CD34 and ERG reactive as well as S100 protein negative [22, 23]. While sharing a similar immunohistochemical profile to our case, histologically these tumors demonstrate a population of bland chondrocyte‐like tumor cells within a chondromyxoid stroma with intervening vascular septa [22]. A distinct round cell population is usually absent. Furthermore, the characteristic fusion of AFCMT (*THBS1::ADGRF5*), though only reported in successfully tested 89% of cases, is a differentiating attribute [22, 23].

Soft tissue chondroma (STC) is a benign neoplasm with an acral predilection, which is microscopically characterized by clusters of cytologically bland chondrocytes within a background matrix of hyaline cartilage and variable myxoid or fibrous areas [24, 25]. The chondrocytes of an STC typically express ERG; however, unlike the present case, they also express S100 protein [25]. Future studies with additional cases would be critical to better understand the relationship between tumors harboring *TCF4::ERG* fusions and other acral mesenchymal neoplasms.

## 5 Conclusion

In summary, we present a case of a CD34‐positive acral chondromyxoid mesenchymal neoplasm with a novel *TCF4::ERG* fusion. This finding contributes to the expanding interplay between surgical pathology and molecular genetics. As a case report, there is limited ability to statistically assess the implications of our observations. If more tumors harboring the *TCF4::ERG* fusion are identified, future studies may investigate demographics, prognosis, histologic characteristics, clinical relevance, and potential therapeutic targets.

## Author Contributions

Eric C. Honaker and Carina A. Dehner conceptualized the study, acquired the data, drafted the manuscript, and provided critical revisions. Laura M. Warmke, Ameline Baptiste, and Daniel Baumhoer contributed to data collection and manuscript editing. Esther Baranov and Eitan Halper‐Stromberg contributed to visualization and figure creation. All authors approved the final manuscript.

## Disclosure

The authors have nothing to report.

## Ethics Statement

In accordance with institutional guidelines, IRB approval was obtained.

## Conflicts of Interest

The authors declare no conflicts of interest.

## Data Availability

The data that support the findings of this study are available on request from the corresponding author. The data are not publicly available due to privacy or ethical restrictions.
